# Envisioning the next steps for machine learning models in integrated cardiovascular-kidney-metabolic care

**DOI:** 10.1093/eschf/xvag045

**Published:** 2026-03-14

**Authors:** Wenze Kan, Mengqi Zhou, Peng Xu

**Affiliations:** School of Clinical Medicine, Jiamusi University, No. 258 Xuefu Street, Xiangyang District, Jiamusi City, Heilongjiang Province 154000, P. R. China; Department of Radiology, The First Affiliated Hospital of Jiamusi University, No. 348 Dexiang Street, Xiangyang District, Jiamusi City, Heilongjiang Province 154000, P. R. China; Department of Nuclear Medicine, Guangzhou Institute of Cancer Research, the Affiliated Cancer Hospital, Guangzhou Medical University, Guangzhou, Guangdong, China; School of Clinical Medicine, Jiamusi University, No. 258 Xuefu Street, Xiangyang District, Jiamusi City, Heilongjiang Province 154000, P. R. China; Department of Radiology, The First Affiliated Hospital of Jiamusi University, No. 348 Dexiang Street, Xiangyang District, Jiamusi City, Heilongjiang Province 154000, P. R. China

**Keywords:** Machine learning, Chronic kidney disease, Worsening heart failure, Electronic health record, Clinical decision support system, Digital twin

Dear Editor,

We read with great interest the recent study by Patel et al.^1^ entitled ‘Clinical Risk Prediction Models for Worsening Heart Failure Events and All-Cause Mortality in Adults with Mild-to-Moderate Chronic Kidney Disease’ published in ESC Heart Failure. The authors developed and validated sophisticated machine learning models using electronic health record (EHR) data, demonstrating excellent predictive performance for worsening heart failure (WHF) and all-cause mortality in this high-risk population. Their work represents a significant advancement in risk stratification for cardiovascular-kidney-metabolic (CKM) syndromes. We wish to extend the discussion on the future clinical translation of such models, focusing on their potential integration with emerging digital health paradigms.

The high discrimination (area under the receiver operating characteristic curve ∼0.88) achieved is commendable. The critical next step is moving from static prediction to dynamic, actionable clinical support. Future iterations could evolve into clinical decision support systems embedded within EHR workflows. These systems could not only flag high-risk patients but also suggest guideline-directed medical therapy (GDMT) optimizations specific to the CKM phenotype, potentially increasing GDMT prescription rates in eligible patients.^2^ The real value lies in closing the loop between risk identification and therapeutic action.

Furthermore, the concept of the digital twin offers a fascinating avenue for extension. A digital twin is a virtual, dynamic model of a patient that simulates health trajectories under different conditions. The predictive framework established by Patel et al. could serve as a foundational component for a CKM digital twin. By integrating their models with time-updated physiological data from wearables or home monitoring devices, one could simulate the long-term impact of various treatment strategies (e.g. SGLT2 inhibitor initiation, dietary adjustments) on an individual's risk of WHF or mortality. This would enable truly personalized, pre-emptive care planning.^3^

A pivotal challenge for deployment, as alluded to by the authors, is data interoperability and real-time computation. Widespread adoption requires models that can operate seamlessly across different EHR architectures. Utilizing Fast Healthcare Interoperability Resources standards for data ingestion and packaging models within scalable, cloud-based platforms will be essential for real-time risk scoring at the point of care.^4^ Additionally, exploring federated learning techniques could allow for model refinement across multiple institutions without sharing sensitive patient data, enhancing generalizability while preserving privacy.

Finally, the application of such predictive tools extends beyond individual patient management to population health. Healthcare systems could use these models to identify subpopulations within their chronic kidney disease cohort that would benefit most from dedicated multidisciplinary CKM clinics or intensive telehealth follow-up, thereby optimizing resource allocation and improving overall care quality.

In conclusion, the study by Patel et al.^1^ provides a powerful predictive engine. Its integration into intelligent clinical support systems, potentially within a digital twin framework, and its implementation via interoperable digital health platforms, heralds a future where predictive analytics directly catalyse personalized, integrated, and proactive CKM care.
